# The Potentiality of Prostate-Specific Antigen as a Prognostic Biomarker in Breast Cancer

**DOI:** 10.7759/cureus.44621

**Published:** 2023-09-03

**Authors:** Wedad Bouaod, Ahmed M Zakoko, Hamza Asif, Azhar Hussain, Nadia Malik, Sidhartha D Ray, Jagannadha Peela, Anirudh Srinivas Teja Peela, Abdalla M Jarari

**Affiliations:** 1 Biochemistry, Benghazi Medical Center, Benghazi, LBY; 2 Medicine, St. George's University - School of Medicine, Princeton, USA; 3 Pharmaceutical and Biomedical Sciences, Touro College of Pharmacy, New York, USA; 4 Biochemistry and Genetics, St. Matthew's University School of Medicine, Grand Cayman, CYM; 5 General Surgery, NRI Institute of Medical Sciences, Visakhapatnam, IND

**Keywords:** free psa, ca19.9, ca125, hematological biomarker, breast cancer, prostate-specific antigen (psa)

## Abstract

Background

Serum prostate-specific antigen (PSA) is a well-established marker that can be measured as an indicator for screening, diagnosing, and managing prostate cancer due to its advanced tissue specificity. Numerous studies have revealed that free PSA is the predominant molecular form of PSA in breast cancer cases. In contrast, total PSA is prevalent in benign breast tumor cases and healthy females. This case-control study aims to measure PSA levels among individuals with breast cancer in order to establish PSA as a prognostic biomarker.

Methods

The study involved 150 female subjects between the ages of 18 and 70 and was conducted between 2013 and 2014. The subjects were then categorized into three groups: those with malignant breast cancer, those with benign breast tumors, and the control group with no history of malignant or benign breast tumors. Participants were asked to complete a lifestyle questionnaire and interview using hospital medical records to establish past and pertinent patient medical history. These cases were acquired from the 7th of October Hospital's surgery department and Benghazi Central Hospital's oncology clinic in Libya. Sandwich-type ELISA’s were used for PSA quantitation, while the Wilcoxon Rank-Sum test was used to identify statistically significant differences between total PSA and free PSA measurements within each patient group.

Results

This study did not reveal significant statistical differences in total PSA levels between breast cancer cases and control groups (p=0.200), or between breast cancer and fibroadenoma patients (p=0.472). However, there was a significant difference in F-PSA levels between breast cancer and fibroadenoma cases (p=0.0001). Neither total-PSA (p=0.200) nor F-PSA (p=0.262) levels showed significant differences between breast cancer cases and controls. This study paved the way for further investigations into PSA's role in breast cancer. Despite its limitations, it offers an opportunity to delve deeper into understanding PSA's potential role and use in breast cancer.

Conclusion

A comprehensive statistical analysis revealed a positive correlation between F-PSA levels and breast cancer diagnosis. The findings suggest that PSA may serve as a prognostic biomarker for breast cancer. This may contribute to improved customized treatment approaches, offering precise and accurate risk assessments, understanding breast cancer biology, and improving health outcomes for patients with breast cancer.

## Introduction

An estimated 13% of females in the general population are predicted to develop breast cancer at some point during their lives, making it one of the biggest medical challenges of our time [1]. Annually, breast cancer affects more than 2.3 million people, making it the most common cancer among adults and the leading cause of cancer death among women [2]. The discovery of new biomarkers for the diagnosis, prognosis, and treatment monitoring of breast cancer has been a major scientific research goal for many years. The discovery of new, highly specific, and/or sensitive biomarkers may aid in developing more effective drugs, providing better prognoses and treatment outcomes for patients suffering from these diseases. For example, prostate-specific antigen (PSA) can be detected in both benign and malignant breast cancers. There are several prognostic factors used to determine the outcomes of breast cancer patients, including lymph node involvement, histological grade, age at diagnosis, estrogen receptor (ER), and progesterone receptor (PR) [3]. PSA are highly specific and have been established as significant tumor markers in prostate cancer. These antigens consist of a serine protease with chymotrypsin-like enzyme activity and consist of two different molecular forms: total PSA (bound form) and free PSA (unbound form) [4]. These antigens inhibit the response of endothelial cells to endothelial growth factor-2 and vascular endothelial growth factor (VEGF) in stimulating angiogenesis, indicating that PSA may function as an endogenous antiangiogenic factor [4-6]. The expression of the PSA gene is under the control of steroid hormones, specifically androgens and progesterone, through the activation of steroid-responsive elements in the promoter/enhancer region [5]. Previous studies have demonstrated that PSA inhibits the proliferation of certain cancer cell lines in vitro by promoting the conversion of potent estradiol to the less potent estrone [6]. Reports indicate that PSA is expressed in 9.3-49% of breast cancers [7, 8]. Evidence exists suggesting its expression in both invasive and noninvasive breast cancers, and this expression might vary based on the histological subtype. Prior research has revealed an inconclusive association between PSA and ER, androgen receptors (AR), and/or the human epidermal growth factor 2 (HER-2) [7-9]. PSA is a non-tissue-specific protein and has been found in a variety of female tissues and body fluids. It has been demonstrated that breasts are one of the most significant female tissues that are capable of producing PSA. Besides normal and abnormal breast tissues, as mentioned above, PSA can also be detected in various breast fluids, including milk, nipple aspirate, and cyst fluid [5, 10-13]. Our preliminary clinical studies indicate that PSA expression may play a role in patient prognosis in breast cancer by affecting ER, PR, tumor size, and clinical stage. A small clinical cohort study found that patients with PSA-positive breast cancer had a significantly lower risk of relapse than those with PSA-negative tumors [14]. PSA production is upregulated by androgen in the prostate through androgen receptors and antagonizes estrogen's effect. Based on this evidence and our preliminary observations, the PSA immunoreactivity may be an indicator of functional steroid hormone receptors and endogenous hormone balance between estrogen, androgen, and progestin in breast cancer cells. Therefore, PSA immunoreactivity in breast tumors could be a useful tool in predicting the prognosis and response to adjuvant therapy in female breast cancer patients [15-18]. To test this hypothesis, we investigated the PSA immunoreactivity in breast cancer associated with relapse-free and overall survival in breast cancer patients. PSA is a valuable tumor marker used for diagnosing and managing prostate cancer. Until recently, PSA was seen as a highly specific biochemical marker of prostatic epithelial cells, and in practice, its detection by immunohistochemistry has been widely used in determining the prostatic origin of metastatic cancer. However, in recent years, PSA has been demonstrated in numerous types of non-prostatic tissues, including parotid glands, kidneys, pancreas, and breast, as well as their malignant counterparts [18]. PSA is a serine protease controlled by one of the three members in the human glandular kallikrein gene family. The other two are tissue kallikrein and human glandular kallikrein-1 [11]. Approximately 60%-80% sequence homology exists between PSA and these two serine proteases. These high levels of homology may explain the positive immunohistochemical reactions for PSA in non-prostatic tissues. The presence of PSA has been shown in normal, hyperplastic, and neoplastic breast tissues. Several reports concerning PSA positivity in breast cancer tissues proposed its utility as a prognostic marker for breast cancer. It has been suggested that the production of PSA in breast tumors is regulated by steroid receptors and is associated with a favorable prognosis [5]. The purpose of this study was to investigate PSA levels in three groups: breast cancer patients, individuals with benign breast disorders, and a control group. The primary goal was to quantitatively assess and analyze PSA levels to establish their viability as a potential biomarker for breast cancer.

## Materials and methods

Ethical approval

The study was registered on August 3, 2013, under approval number 36/2022 by the Institutional Review Board of Benghazi University Medical Center. In order to ensure the confidentiality of the data, all personal identifying information was removed, and only medically significant parameters were analyzed.

Study participants

The study involved 150 female participants, who were subsequently categorized into three groups. The first group included 76 women diagnosed with breast cancer between the ages of 30 and 70. The second group included 22 women with benign breast disorders such as fibroadenomas between the ages of 18 and 50. The third group served as the control group for the study and included 52 participants between the ages of 18 and 56, all free of malignant and benign breast disorders. The inclusion criteria for the breast cancer and fibroadenoma groups included females between the ages of 18 and 80 from diverse racial and ethnic backgrounds, those with histopathologically confirmed breast cancer, malignancy of any stage (I, II, III, and IV), and those who had undergone surgical procedures. The control group included females between ages 18 and 80 from diverse ethnic and racial backgrounds and individuals with any prior history of carcinoma. Participants who were pregnant, had psychiatric impairments or were unwilling or unable to provide informed consent were excluded. The sample size was determined using data collected from patients who fit the study's inclusion criteria and were examined at the 7th of October Hospital’s surgery department and Benghazi Central Hospital’s oncology clinic during 2013 and 2014. Informed consent was obtained from each participant. Each participant completed a lifestyle questionnaire, which included questions regarding reproductive history, ages at menarche, menopause, age of first birth, parity, and family history of breast cancer. Participants were also interviewed to obtain patients' histories, which were confirmed and compared with existing medical records and hospital files. A review of breast cancer risk factors, medical history, and exogenous hormone use was also conducted. 

Sample collection and analysis

Each patient had a venous arm puncture, and 5 ml of blood was collected and then immediately transferred to a clean, dry, plain tube. Blood was allowed to clot at room temperature for at least 10-15 minutes after removing the needle. The serum was then separated by centrifugation at 3000 rpm for 15 minutes. The samples were then carefully transferred into plastic tubes and stored at 2-8°C for 24 hours before being tested. Serum samples were either analyzed immediately or stored at -35ºC until they were analyzed for further use. The serum which was removed was used for the measurement of hormonal and biochemical parameters. All work was conducted in the Hormones and Biochemistry Department of Al-Gomhorryia Hospital, located in Benghazi, Libya. PSA measurements were quantified using sandwich-type enzyme-linked immunosorbent assay (ELISA) by Roche Diagnostics, while Wilcoxon Rank-Sum tests were also used to detect statistically significant differences between total PSA and free PSA measurements. Serum tumor markers CA125, CA15.3, and carcinoembryonic antigen (CEA) levels were measured by authenticated methods using a Cobas E411 analyzer. Estradiol II immunoassay test kits were used for estimation, while a Cobas E411 analyzer was used to quantitatively determine estradiol (E2) concentration in human sera. A calibration curve was used to determine the final results.

## Results

Out of those total 150 subjects, there were 76 breast cancer cases with an average age of 48 years, controls with an average age of 41 years, and fibroadenosis cases with an average age of 31 years. The average weight of a patient with breast cancer was 78.3 kilograms (kg), compared to 77.34 kg for a control patient and 72.86 kg for a patient with fibroadenosis. The average height of a patient with breast cancer was 1.6 meters, that of a control patient was 1.63 meters, and that of a patient with fibroadenosis was 1.64 meters, as shown in Table 1.

**Table 1:** Demographic data of participants.

	Age	Weight (Kgs)	Height (Meters)	BMI (kg/m^2^)
Breast Cancer Cases	48 ± 11	78.3 ± 14.7	1.6 ± 0.1	31.05 ± 5.1
Controls	41.38 ± 9.61	77.34 ± 12.04	1.63 ± 0.076	25.48 ± 5.4
Fibroadenosis Cases	31.62 ± 11.38	72.86 ± 12.78	1.64 ± 0.03	27.15 ± 6.7

For each group of subjects, we analyzed the mean and SD for cancer antigens (CA) 125, 15.3, 19.9, CEA, estradiol (E2), total PSA, and free PSA values. The mean serum levels of CA125 (18.0 U/mL (±13.8 U/mL)) and CA15.3 (18.9 U/mL (±24.6 U/mL)) in the cases were not significantly elevated compared to the controls (14.16 U/mL (±10.25 U/mL)) with p-values of 0.1525 and 0.1004, respectively. No significant difference was observed in CEA levels between the controls and breast cancer cases (p=0.23). The serum levels of E2 (41.9 pg/mL (±26.1 pg/mL)) were significantly lower in breast cancer cases compared to the controls (119.51 pg/mL (±99.59 pg/mL)) with a p-value of 0.0001. No significant differences were noted in total-PSA (p=0.200) and F-PSA (p=0.262) values between cases and controls. Detailed results are presented in Tables 2-3, respectively.

**Table 2:** Mean, SD, and p-value levels amongst breast cancer, fibroadenoma, and control groups. CA-125: Cancer antigen 125; CA-15.3: Cancer antigen 15.3; CA-19.9: Cancer antigen 19.9; CEA: Carcinoembryonic antigen; E2: Estradiol.

Parameters	Breast Cancer Cases (76)				Fibroadenoma Cases (21)			Controls (52)	
	Mean	SD	P-value (Breast Cancer/Controls)	P-value (Breast Cancer/Fibroadenomas)	Mean	SD	P-value	Mean	SD
CA125 (U/mL)	18.0	13.8	0.1525	0.0195	11.0	6.70	0.1971	14.16	10.25
CA15.3 (U/mL)	18.9	24.6	0.1004	0.1891	11.91	4.25	0.4857	13.01	6.65
CA19.9 (U/mL)	13.9	13.9	0.0348	0.0144	6.60	6.00	0.1454	9.37	7.72
CEA (ng/mL)	2.0	2.8	0.23	0.2651	1.31	0.46	0.2315	1.52	0.74
E2 (pg/mL)	41.9	26.1	0.0001	0.0001	135.99	105.24	0.5309	119.51	99.59

**Table 3:** Total and free PSA levels amongst breast cancer, fibroadenoma, and control groups. Total PSA: Total prostate-specific antigen; F-PSA: Free prostate-specific antigen.

Parameters	Breast Cancer Cases (76)				Fibroadenoma Cases (21)			Controls (52)	
	Mean	SD	P-value (Breast Cancer/Controls)	P-value (Breast Cancer/Fibroadenomas)	Mean	SD	P-value	Mean	SD
Total PSA (ng/mL)	0.059	0.273	0.200	0.472	0.006	0.009	0.6922	0.0033	0.0265
F-PSA (ng/mL)	0.026	0.062	0.262	0.0001	0.013	0.014	0.0001	0.02	0.0865

The serum levels of CA125 in fibroadenoma cases (11.0 U/mL (±6.70 U/mL)) were not significantly different from the controls [14.16 U/mL (±10.25 U/mL)] with a p-value of 0.4857. CA15.3 levels in fibroadenoma cases (11.91 U/mL (±4.25 U/mL)) were comparable to the controls (13.01 U/mL (±6.65 U/mL)) with a p-value of 0.1454. There was no statistically significant difference in CEA levels between the controls and the breast cancer cases (p=0.2315). Similarly, no significant differences were observed in E2 (p=0.5309) and total PSA levels (p=0.6922). A statistically significant decrease in the F-PSA was observed between the control group and fibroadenoma cases (Tables 2 and 3). When we compared breast cancer and fibroadenoma cases, there was a statistically significant difference between CA125 (p=0.0195) and CA19.9 (p=0.0144). Both CA15.3 (p=0.1891) and CEA (p=0.2651) showed no statistically significant difference between breast cancer and fibroadenoma cases. Regarding E2 levels, there was a statistically significant decline in breast cancer cases (p=0.0001). The total PSA levels were similar between breast cancer and fibroadenoma cases (p=0.4719). Remarkably, the concentration of F-PSA was notably elevated in fibroadenoma cases (0.013 ng/mL (±0.014 ng/mL)) when compared to breast cancer cases (0.0264 ng/mL (±0.06249 ng/mL)), (p=0.0001).

## Discussion

Breast cancer patients would benefit greatly from tools that can independently predict their recurrence rate in primary breast cancer and their response to systemic therapy. The presence of steroid receptors in the primary tumor does not completely predict whether patients with recurrent breast cancer will benefit from endocrine therapy. In some cases, anti-estrogen treatment is ineffective for some patients with steroid receptor-positive tumors [19]. Therefore, refining therapy tailored to the patient's needs may benefit from biological factors other than estrogen and progesterone receptors (PgR). Primary breast cancers with aberrant expression of specific cell parameters associated with a high risk of relapse do not necessarily warrant adjuvant treatment. It is possible for a particular form of systems therapy to be inactive or even stimulate tumor growth by triggering the cell biological factor involved. For this reason, biological parameters should be evaluated in both primary and metastatic breast cancer to determine their prognostic value. Several factors can influence prognosis in primary breast cancer, including the serine protease PSA, whose expression is mediated by steroid hormones. It has been suggested that PSA expression might be linked to responses to endocrine therapy [17]. Baseline demographics such as age, BMI, and other associated factors might introduce bias, potentially skewing the results obtained from the chosen sample size. However, further research is required to establish any correlations between these variables and the study's outcomes. Previous reports of prostatic adenocarcinoma metastatic to the breast have emphasized the usefulness of prostatic markers in distinguishing it from primary breast cancer. However, recent evidence of PSA expression in female breast cancers calls for caution, as underscored by our current study. The breast contains hormone receptors responsive to estrogen, progesterone, and androgen. While estrogen can inhibit PSA expression, androgens can stimulate it. The steroid hormone estrogen plays a pivotal role in the development and progression of breast cancer. Hence, depleting, counteracting, and diminishing estrogenic stimuli could potentially be beneficial in the treatment of breast cancer and breast cancer management. Research has shown that androgens exhibit an anti-estrogenic impact, thus impeding the proliferation of breast cancer cells [20]. In actuality, androgens have been utilized as a successful treatment modality in individuals with breast cancer [21, 22]. Because the expression of PSA is steroid-meditated, the presence of PSA immunoreactivity within breast cancer cells could potentially indicate endogenous hormonal equilibrium between estrogen and androgen/progestin [20]. Hence, positively identifying PSA immunoreactivity in breast tumors may be invaluable in forecasting the prognosis of breast cancer patients. In fact, PSA expression may indicate a favorable outcome.

PSA, a member of the kallikrein family, digests semenogelins and fibronectin, both of which are found at elevated levels in seminal plasma. This process liquefies the seminal clot shortly after ejaculation [23]. The role of PSA in cancer is still a matter of debate, with studies suggesting both procarcinogenic and anti-carcinogenic properties. PSA also acts as a growth regulator by cleaving insulin-like growth factor binding protein-3 (IGFBP-3), either releasing insulin-like growth factor-I (a mitogen) or enzymatically activating latent human transforming growth factor-alpha [24, 25]. PSA has been linked to degrading extracellular matrix proteins- fibronectin and laminin - and may play a role in tumor progression and metastasis. PSA further triggers the activation of transforming growth factor-ß (TGF-ß), promotes cell detachment, and aids in disseminating tumors [25, 26]. The presence of PSA expression in well-differentiated tumors offers a survival advantage, as these tumors tend to respond better to treatment and generally have a more favorable prognosis compared to poorly differentiated cancers. PSA proteolytically cleaves parathyroid hormone-related proteins (PTHrP), which stimulate breast cancer cell proliferation, thereby neutralizing its biological function [27]. Moreover, PSA has been shown to exert a suppressive effect on the endothelial response to angiogenic stimuli from fibroblast growth factor-2 and vascular endothelial growth factor. By digesting plasminogen, PSA releases antiangiogenic fragments (similar to angiostatin) and promotes the conversion of the more potent estradiol to the less potent estrone, thus mitigating the procarcinogenic effects of estrogen [28]. Using an ultrasensitive time-resolved immunofluorometric assay for PSA, we found that 30% of female breast tumor cytosols from a cohort of more than 1,200 breast cancer patients contained PSA immunoreactivity higher than 0.03 ng/mg of total protein [6, 16]. Such immunoreactivity was also detected by three commercially available PSA kits. The molecular weight of PSA immunoreactivity in breast tumors measured by high-performance liquid chromatography (HPLC) and Western Blot techniques was identical to the molecular weight of PSA in seminal plasma [17]. We further found that PSA immunoreactivity in breast cancer cells was associated with ER, PR, and the early clinical stage [16, 17]. Finally, our cell culture studies demonstrated that androgens or progestins could induce breast cancer cell lines T-470 and MCF-7 to produce PSA immunoreactivity and that this induction was suppressed by estrogen. Tamoxifen, an anti-estrogen agent, also induces PSA production in these cell lines [18]. The production of PSA immunoreactivity was observed only in breast cancer cell lines with steroid hormone receptors (T-470 and MCF-7 cells). No product was seen for the breast cancer cell line BT-20, which does not possess steroid hormone receptors [8, 29]. Decisions regarding whether and how to treat breast cancer patients after surgery significantly influence both patient survival and healthcare costs. It's crucial to rationally review prognostic parameters before making these decisions [29]. Current prognostic and predictive markers are a blend of host factors (e.g., menopausal status, inflammatory response) and tumor characteristics (e.g., tumor size, histological grade, cell proliferation) [4, 5, 10, 11, 15]. Despite the broad array of available markers, physicians often struggle to distinguish patients who will benefit from adjuvant treatment from those who will not, largely due to the markers' lack of sensitivity and specificity. The end objective is to ultimately use prognostic and predictive markers to allow physicians to accurately differentiate between patients requiring post-surgical treatment and appropriately tailor treatment to their specific needs.

Limitations

The first limitation of this study is that it was solely conducted in Libya. This may cause the study to exhibit limited variability within the data collected and not reflect the global population. A second limitation exhibited pertained to the modest sample size of participants, which amounted to 150 individual cases. This may reduce the study's statistical power and potentially introduce large margins of error in the data and subsequent findings. A third limitation of the study was the inclusion of cases from just two hospitals. Obtaining data from two hospitals does not introduce enough variability within the study and may be skewed due to the limited medical practices, demographics, and disease prevalence surrounding the area in which the hospitals are located. Conducting the study in one country captures its limitations, whereas incorporating data from a large range of hospitals would have offered a more comprehensive analysis of the country's entire population. A final limitation of the study was the deviated age ranges within the three groups of participants. While the age of one group ranged from 30 to 70, the others ranged between 18-50 and 18-56. This may present issues in findings as there may be a potential interaction between age and presented outcomes.

## Conclusions

Statistically significant differences were observed in F-PSA levels between breast cancer cases and fibroadenoma cases. This suggests that prostate serum antigens might serve as potential diagnostic, prognostic, and monitoring tools for breast cancer patients. However, given the limitations of the study, further investigations are necessary to evaluate PSA's role in breast cancer. Such exploration holds the potential to improve health outcomes, deepen understanding of breast cancer biology, and pave the way for the development of new and effective drugs and treatments.
